# Item

**Published:** 1897-10

**Authors:** 


### Item.

The American Pediatric Society is making a collective investigation of infantile scurvy, as occurring in North America, and earnestly requests the coöperation of physicians in sending reports of cases whether they have already been published or not. No case will be used in such a way as to interfere with its subsequent publication by the observer. Blanks containing questions to be filled out will be furnished on application to any one of the following-named committee. A final printed report of the investigation will be sent to those furnishing cases. J. P. Crozer Griffith, M. D., chairman, 123 South 18th street, Philadelphia; William D. Booker, M. D., 853 Park avenue, Baltimore; Charles G. Jennings, M. D., 457 Jefferson avenue, Detroit; Augustus Caille, M. D., 753 Madison avenue, New York City; J. Lovett Morse, M. D., 317 Marlboro street, Boston.

